# 4-Bromo-2-[({2-[(2-hy­droxy­eth­yl)amino]­eth­yl}imino)­meth­yl]phenol

**DOI:** 10.1107/S2414314621003357

**Published:** 2021-04-09

**Authors:** Erika Samoľová, Aliakbar Dehno Khalaji, Václav Eigner

**Affiliations:** a Institute of Physics AS CR, v.v.i., Na Slovance 2, 182 21 Prague 8, Czech Republic; b Department of Chemistry, Faculty of Science, Golestan University, Gorgan, Iran; Goethe-Universität Frankfurt, Germany

**Keywords:** crystal structure, Schiff base, hydrogen bonds

## Abstract

The new title Schiff base compound, C_11_H_15_BrN_2_O_2_, crystallizes in the monoclinic space group *P*2_1_ with two independent mol­ecules in the asymmetric unit. It was prepared by the condensation reaction of 5-bromo-2-hy­droxy­benzaldehyde and amino­ethyl­ethano­lamine. There is an intra­molecular O—H⋯N hydrogen bond with an *S*(6) ring motif. Moreover, there are inter­molecular C—H⋯N, C—H⋯O and Br⋯O inter­actions in the crystal structure of the title compound.

## Abstract 2

The synthesis and crystal structure of a new Schiff base, namely, 4-bromo-2-[({2-[(2-hy­droxy­eth­yl)amino]­eth­yl}imino)­meth­yl]phenol, is reported.

## Graphical abstract

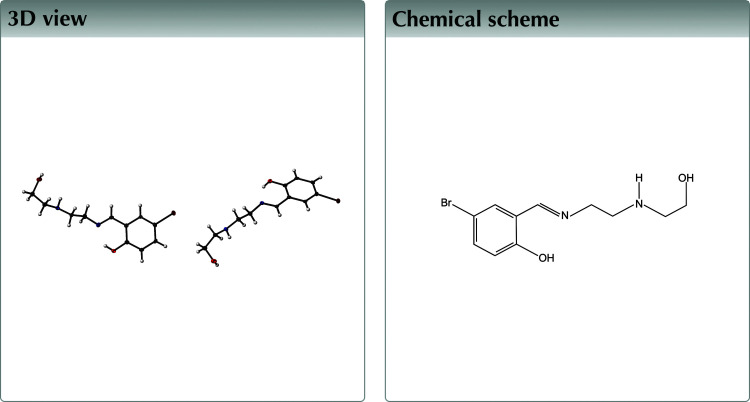

## Structure description

**Figure 1:**
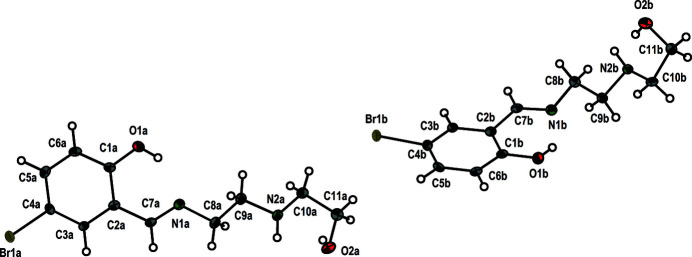
The asymmetric unit of the title structure. Displacement ellipsoids are drawn at the 50% probability level.

**Figure 2:**
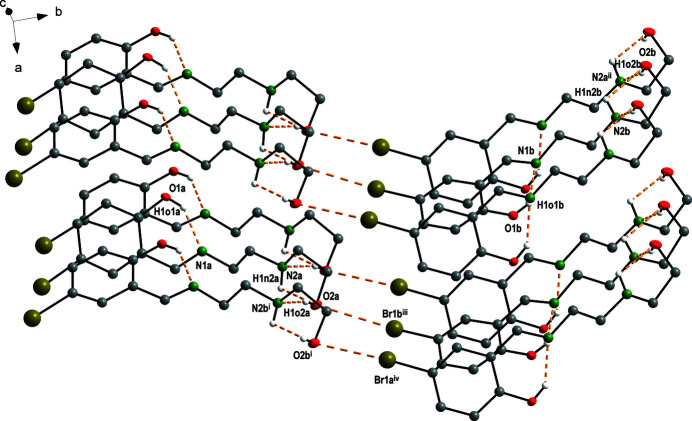
View of the hydrogen-bond system and the Br⋯O inter­actions in the title compound. Hydrogen atoms not involved in hydrogen bonding are omitted for clarity. Symmetry codes: (i) 2 − *x*, *y* − 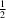 , 2 − *z*; (ii) 1 − *x*, 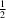 + *y*, 1 − *z*; (iii) 1 + *x*, *y*, *z*; (iv) 3 − *x*, 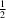 + *y*, 2 − *z*.

Schiff bases and their derivatives have played a key role in the development of coordination chemistry (Vafaza­deh *et al.*, 2019 ▸; Ghorbani *et al.*, 2017 ▸) due to their easy preparation, structural diversity, biological properties, catalytic activity and also their ability to act as chelating ligands (Böhme & Fels, 2020 ▸; Adrian *et al.*, 2020 ▸; Saranya *et al.*, 2020 ▸; Yousif *et al.*, 2017 ▸; Guo *et al.*, 2019 ▸; Bhattacharjee *et al.*, 2017 ▸; Shweta *et al.*, 2016 ▸; Reimann *et al.*, 2019 ▸; Ceylan *et al.*, 2015 ▸; Salehi *et al.*, 2016 ▸; Zhu *et al.*, 2019 ▸; Kumar *et al.*, 2019 ▸; Atahan & Durmus, 2015 ▸). In the present work, we report the crystal structure of the new Schiff base, commonly known as amino­ethyl­ethano­lamine-5-bromo-2-hy­droxy­benzaldehyde. The asymmetric unit of the title compound contains two independent mol­ecules, as shown in Fig. 1 ▸. All bond lengths and angles are within their expected ranges according to other published Schiff base structures (Böhme & Fels, 2020 ▸; Ceylan *et al.*, 2015 ▸; Salehi *et al.*, 2016 ▸). The N1*a*=C7*a* double bond is 1.273 (6) Å and N1*b*=C7*b* 1.276 (7) Å, the N1*a*—C8*a* single bond is 1.460 (6) Å and N1*b*—C8*b* 1.455 (7) Å, in good agreement with the corresponding values for the similar compounds (Salehi *et al.*, 2016 ▸; Ceylan *et al.*, 2015 ▸). The bond angles C7*a*—N1*a*—C8*a* [118.0 (4)°], C7*b*—N1*b*—C8*b* [117.9 (4)°], C2*a*—C7*a*—N1*a* [121.5 (4)°] and C2*b*—C7*b*—N1*b* [121.7 (4)°] are also in agreement with those angles in the similar compounds (Salehi *et al.*, 2016 ▸; Ceylan *et al.*, 2015 ▸). An intra­molecular hydrogen bond with an *S*(6) ring is observed in each independent mol­ecule. Moreover, the O2*a* and O2*b* atoms are involved in a second intra­molecular hydrogen bond. The mol­ecules are connected through inter­molecular O—H⋯N hydrogen bonds and Br⋯O inter­actions with distances Br1*a*⋯O2*b* = 3.206 (2) Å and Br1*b*⋯O2*a* = 3.282 (2) Å (Fig. 2 ▸, Table 1 ▸).

## Synthesis and crystallization

5-Bromo-2-hy­droxy­benzaldehyde (2 mmol) was dissolved in ethanol (10 ml) and stirred for 10 min. Then, a solution of amino­ethyl­ethano­lamine (0.2 mmol) dissolved in ethanol (5 ml) was added dropwise. The mixture was stirred and refluxed for 6 h. After that, the solution was concentrated under reduced pressure. Yellow crystals suitable for X-ray analysis were obtained by slow evaporation of solvent at room temperature for several days. These were filtered off and washed several times with cold ethanol.

## Refinement

Crystal data, data collection and structure refinement details are summarized in Table 2 ▸. Marching Cube ELD software (MCS) was used for the electron density map visualization (Rohlíček & Hušák, 2007 ▸).

## Supplementary Material

Crystal structure: contains datablock(s) global, I. DOI: 10.1107/S2414314621003357/bt4109sup1.cif

Structure factors: contains datablock(s) I. DOI: 10.1107/S2414314621003357/bt4109Isup2.hkl

Supporting information file. DOI: 10.1107/S2414314621003357/bt4109Isup3.cml

CCDC reference: 2074082

Additional supporting information:  crystallographic information; 3D view; checkCIF report

## Figures and Tables

**Table 1:** Hydrogen-bond geometry (Å, °)

*D*—H⋯*A*	*D*—H	H⋯*A*	*D*⋯*A*	*D*—H⋯*A*
O1*a*—H1*o*1*a*⋯N1*a*	0.82 (5)	1.89 (6)	2.597 (5)	144 (5)
O2*a*—H1*o*2*a*⋯N2*b* ^i^	0.82 (3)	2.02 (4)	2.826 (6)	168 (6)
O1*b*—H1*o*1*b*⋯N1*b*	0.82 (5)	1.91 (6)	2.587 (6)	139 (5)
O2*b*—H1*o*2*b*⋯N2*a* ^ii^	0.82 (3)	2.06 (4)	2.859 (6)	165 (6)
N2*a*—H1*n*2*a*⋯O2*a*	0.88 (3)	2.45 (5)	2.871 (6)	110 (5)
N2*b*—H1*n*2*b*⋯O2*b*	0.88 (3)	2.44 (5)	2.884 (5)	112 (5)

Symmetry codes: (i) 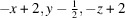 ; (ii) 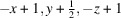 .

**Table 2:** Experimental details

Crystal data	
Chemical formula	C_11_H_15_BrN_2_O_2_
*M*_r_	287.2
Crystal system, space group	Monoclinic, *P*2_1_
Temperature (K)	95
*a*, *b*, *c* (Å)	6.0518 (2), 27.7837 (7), 6.9028 (2)
β (°)	90.497 (2)
*V* (Å^3^)	1160.60 (6)
*Z*	4
Radiation type	Cu *K*α
μ (mm^−1^)	4.74
Crystal size (mm)	0.38 × 0.25 × 0.04

Data collection	
Diffractometer	Rigaku Oxford Diffraction SuperNova, Dual, Cu at zero, AtlasS2
Absorption correction	Analytical (*CrysAlis PRO*; Rigaku OD, 2015 ▸)
*T*_min_, *T*_max_	0.34, 0.816
No. of measured, independent and observed [*I* > 3σ(*I*)] reflections	8118, 4637, 4488
*R*_int_	0.021
(sin θ/λ)_max_ (Å^−1^)	0.628

Refinement	
*R*[*F* ^2^ > 2σ(*F* ^2^)], *wR*(*F* ^2^), *S*	0.055, 0.134, 2.31
No. of reflections	4637
No. of parameters	308
No. of restraints	6
H-atom treatment	H atoms treated by a mixture of independent and constrained refinement
Δρ_max_, Δρ_min_ (e Å^−3^)	0.81, −0.48
Absolute structure	Flack (1983 ▸), 2215 Friedel pairs
Absolute structure parameter	−0.03 (3)

Computer programs: *CrysAlis PRO* (Rigaku OD, 2015 ▸), *SUPERFLIP* (Palatinus & Chapuis, (2007 ▸), *JANA2006* (Petříček *et al.*, 2014 ▸), *MCE* (Rohlíček & Hušák, 2007 ▸) and *DIAMOND* (Brandenburg, 1999 ▸).

## full crystallographic data

### Crystal data

**Table:**

C_11_H_15_BrN_2_O_2_	*F*(000) = 584
*M**_r_* = 287.2	*D*_x_ = 1.643 Mg m^−^^3^
Monoclinic, *P*2_1_	Cu *K*α radiation, λ = 1.54184 Å
Hall symbol: P 2yb	Cell parameters from 5986 reflections
*a* = 6.0518 (2) Å	θ = 6.4–75.4°
*b* = 27.7837 (7) Å	µ = 4.74 mm^−^^1^
*c* = 6.9028 (2) Å	*T* = 95 K
β = 90.497 (2)°	Platelet, colourless
*V* = 1160.60 (6) Å^3^	0.38 × 0.25 × 0.04 mm
*Z* = 4	

### Data collection

**Table:**

Rigaku Oxford Diffraction SuperNova, Dual, Cu at zero, AtlasS2 diffractometer	4637 independent reflections
Radiation source: X-ray tube	4488 reflections with *I* > 3σ(*I*)
Mirror monochromator	*R*_int_ = 0.021
Detector resolution: 5.2027 pixels mm^-1^	θ_max_ = 75.5°, θ_min_ = 6.4°
ω scans	*h* = −7→7
Absorption correction: analytical (CrysAlisPro; Rigaku OD, 2015)	*k* = −34→34
*T*_min_ = 0.34, *T*_max_ = 0.816	*l* = −7→8
8118 measured reflections	

### Refinement

**Table:**

Refinement on *F*^2^	H atoms treated by a mixture of independent and constrained refinement
*R*[*F*^2^ > 2σ(*F*^2^)] = 0.055	Weighting scheme based on measured s.u.'s *w* = 1/(σ^2^(*I*) + 0.0016*I*^2^)
*wR*(*F*^2^) = 0.134	(Δ/σ)_max_ = 0.0004
*S* = 2.31	Δρ_max_ = 0.81 e Å^−^^3^
4637 reflections	Δρ_min_ = −0.48 e Å^−^^3^
308 parameters	Absolute structure: Flack (1983), 2215 Friedel pairs
6 restraints	Absolute structure parameter: −0.03 (3)
103 constraints	

### Special details

**Table:**

Refinement. All hydrogen atoms were discernible in difference Fourier maps and could be refined to reasonable geometry. Marching Cube ELD software (MCS) was used for the electron density map visualization (Rohlicek & Husak, 2007). According to common practice, H atoms bonded to C were kept in ideal positions with C–H = 0.96 Å while positions of H atom bonded to N and O were refined with restrained bond lengths 0.820 (1) Å for O—H bonds and 0.880 (1) Å for N—H bonds. In both cases *U*_iso_(H) was set to 1.2*U*_eq_(C,*N*,*O*). All non-hydrogen atoms were refined using harmonic refinement.

### Fractional atomic coordinates and isotropic or equivalent isotropic displacement parameters (Å^2^)

**Table:**

	*x*	*y*	*z*	*U*_iso_*/*U*_eq_	
Br1a	0.82327 (7)	0.119124 (17)	0.59379 (6)	0.01689 (14)	
Br1b	0.67219 (7)	0.617268 (17)	0.82063 (6)	0.01663 (14)	
O1a	0.3912 (6)	0.31366 (13)	0.6749 (5)	0.0160 (10)	
O2a	1.4292 (6)	0.51220 (14)	0.8603 (5)	0.0181 (10)	
O1b	1.0984 (6)	0.81041 (13)	0.9575 (6)	0.0170 (10)	
O2b	0.0585 (6)	1.01653 (14)	0.6475 (5)	0.0173 (10)	
N1a	0.7625 (7)	0.35173 (15)	0.5676 (6)	0.0136 (11)	
N2a	1.0395 (7)	0.46689 (15)	0.7058 (6)	0.0138 (11)	
N1b	0.7210 (7)	0.84968 (16)	0.8774 (6)	0.0147 (11)	
N2b	0.4536 (7)	0.96878 (15)	0.7831 (6)	0.0130 (11)	
C1a	0.4889 (8)	0.27034 (18)	0.6505 (7)	0.0123 (13)	
C2a	0.7071 (8)	0.26658 (17)	0.5792 (6)	0.0113 (12)	
C3a	0.8022 (7)	0.22102 (17)	0.5563 (7)	0.0110 (12)	
C4a	0.6828 (8)	0.18042 (17)	0.6059 (7)	0.0127 (13)	
C5a	0.4692 (8)	0.18358 (18)	0.6760 (7)	0.0135 (13)	
C6a	0.3746 (8)	0.22869 (18)	0.6977 (7)	0.0136 (13)	
C7a	0.8414 (8)	0.30963 (18)	0.5459 (7)	0.0119 (12)	
C8a	0.9119 (8)	0.39261 (18)	0.5457 (8)	0.0153 (14)	
C9a	0.9002 (8)	0.42429 (17)	0.7262 (7)	0.0156 (13)	
C10a	1.0340 (9)	0.49712 (19)	0.8800 (7)	0.0147 (14)	
C11a	1.2178 (9)	0.53440 (19)	0.8765 (8)	0.0169 (14)	
C1b	1.0028 (8)	0.76776 (19)	0.9183 (7)	0.0132 (13)	
C2b	0.7840 (8)	0.76505 (17)	0.8468 (7)	0.0123 (13)	
C3b	0.6901 (7)	0.71945 (18)	0.8096 (6)	0.0111 (12)	
C4b	0.8125 (8)	0.67826 (17)	0.8475 (7)	0.0128 (13)	
C5b	1.0266 (8)	0.68081 (17)	0.9183 (7)	0.0129 (13)	
C6b	1.1215 (8)	0.72560 (19)	0.9527 (7)	0.0138 (13)	
C7b	0.6447 (8)	0.80791 (19)	0.8387 (7)	0.0131 (13)	
C8b	0.5641 (8)	0.8890 (2)	0.8969 (7)	0.0174 (15)	
C9b	0.5974 (8)	0.92782 (17)	0.7427 (7)	0.0132 (12)	
C10b	0.4570 (8)	1.00481 (18)	0.6268 (7)	0.0140 (14)	
C11b	0.2681 (8)	1.04018 (18)	0.6529 (8)	0.0166 (14)	
H1o1a	0.477 (9)	0.3360 (17)	0.656 (10)	0.0192*	
H1o2a	1.447 (12)	0.501 (2)	0.970 (4)	0.0217*	
H1o1b	1.022 (10)	0.8341 (15)	0.933 (10)	0.0204*	
H1o2b	0.047 (12)	1.006 (2)	0.537 (4)	0.0208*	
H1n2a	1.177 (3)	0.460 (2)	0.679 (9)	0.0165*	
H1n2b	0.316 (4)	0.958 (2)	0.786 (9)	0.0157*	
H1c3a	0.949114	0.217962	0.506473	0.0132*	
H1c5a	0.388396	0.155044	0.708997	0.0162*	
H1c6a	0.226862	0.231176	0.746291	0.0163*	
H1c7a	0.992328	0.305995	0.506916	0.0142*	
H1c8a	0.868714	0.41104	0.433975	0.0184*	
H2c8a	1.06028	0.381103	0.530093	0.0184*	
H1c9a	0.750008	0.434045	0.7466	0.0187*	
H2c9a	0.947685	0.406141	0.837289	0.0187*	
H1c10a	1.050579	0.477245	0.992939	0.0176*	
H2c10a	0.893641	0.513099	0.887122	0.0176*	
H1c11a	1.194585	0.555849	0.769179	0.0203*	
H2c11a	1.213354	0.553276	0.992845	0.0203*	
H1c3b	0.542552	0.716952	0.758373	0.0133*	
H1c5b	1.108922	0.65191	0.943463	0.0155*	
H1c6b	1.270528	0.727504	1.00081	0.0166*	
H1c7b	0.491711	0.804593	0.8034	0.0157*	
H1c8b	0.579114	0.903103	1.023326	0.0208*	
H2c8b	0.41626	0.876651	0.887421	0.0208*	
H1c9b	0.748732	0.938194	0.744645	0.0159*	
H2c9b	0.561579	0.91489	0.617336	0.0159*	
H1c10b	0.441354	0.988939	0.503918	0.0168*	
H2c10b	0.59503	1.021837	0.630526	0.0168*	
H1c11b	0.285747	1.056606	0.77445	0.0199*	
H2c11b	0.273235	1.0641	0.552719	0.0199*	

### Atomic displacement parameters (Å^2^)

**Table:**

	*U*^11^	*U*^22^	*U*^33^	*U*^12^	*U*^13^	*U*^23^
Br1a	0.0181 (2)	0.0121 (2)	0.0204 (2)	0.0014 (2)	−0.00076 (17)	−0.0019 (2)
Br1b	0.0185 (2)	0.0122 (2)	0.0192 (2)	−0.0015 (2)	−0.00028 (17)	−0.0015 (2)
O1a	0.0109 (16)	0.0154 (17)	0.0216 (18)	0.0020 (13)	0.0010 (14)	−0.0024 (15)
O2a	0.0133 (17)	0.0247 (19)	0.0162 (17)	−0.0031 (14)	0.0002 (14)	0.0033 (15)
O1b	0.0106 (16)	0.0154 (17)	0.0249 (19)	−0.0008 (13)	−0.0014 (14)	−0.0021 (15)
O2b	0.0138 (16)	0.0240 (19)	0.0143 (16)	0.0014 (14)	−0.0011 (13)	−0.0026 (14)
N1a	0.0119 (18)	0.015 (2)	0.0133 (18)	−0.0008 (16)	−0.0012 (14)	0.0011 (15)
N2a	0.0125 (18)	0.0143 (19)	0.0146 (19)	−0.0025 (15)	0.0002 (15)	0.0001 (16)
N1b	0.0149 (19)	0.014 (2)	0.015 (2)	0.0022 (16)	0.0014 (16)	0.0023 (16)
N2b	0.0105 (18)	0.0136 (19)	0.015 (2)	−0.0003 (14)	0.0009 (15)	−0.0015 (16)
C1a	0.011 (2)	0.017 (2)	0.009 (2)	0.0011 (18)	−0.0007 (17)	−0.0011 (18)
C2a	0.011 (2)	0.017 (2)	0.0064 (19)	−0.0010 (18)	−0.0020 (16)	0.0004 (17)
C3a	0.008 (2)	0.013 (2)	0.0116 (19)	−0.0002 (17)	−0.0007 (16)	0.0009 (17)
C4a	0.015 (2)	0.011 (2)	0.011 (2)	0.0018 (17)	−0.0019 (17)	−0.0020 (16)
C5a	0.014 (2)	0.016 (2)	0.010 (2)	−0.0047 (18)	−0.0037 (18)	0.0009 (17)
C6a	0.008 (2)	0.022 (3)	0.011 (2)	−0.0001 (19)	−0.0018 (17)	−0.0017 (18)
C7a	0.009 (2)	0.016 (2)	0.010 (2)	−0.0003 (17)	−0.0003 (16)	0.0014 (18)
C8a	0.013 (2)	0.014 (2)	0.019 (3)	−0.0026 (18)	−0.0001 (18)	0.0023 (18)
C9a	0.016 (2)	0.015 (2)	0.016 (2)	−0.0021 (18)	0.0002 (18)	−0.0004 (18)
C10a	0.012 (2)	0.017 (3)	0.016 (2)	0.0000 (19)	−0.0005 (19)	0.0000 (18)
C11a	0.016 (2)	0.019 (3)	0.016 (2)	0.0004 (19)	−0.0018 (19)	−0.0006 (19)
C1b	0.012 (2)	0.017 (2)	0.010 (2)	−0.0011 (18)	0.0033 (17)	−0.0001 (18)
C2b	0.013 (2)	0.015 (2)	0.008 (2)	0.0004 (18)	0.0011 (17)	0.0010 (18)
C3b	0.009 (2)	0.013 (2)	0.011 (2)	0.0007 (17)	−0.0004 (16)	0.0014 (17)
C4b	0.016 (2)	0.013 (2)	0.010 (2)	−0.0021 (18)	0.0012 (18)	−0.0023 (17)
C5b	0.015 (2)	0.013 (2)	0.011 (2)	0.0054 (18)	0.0016 (18)	0.0005 (17)
C6b	0.009 (2)	0.019 (2)	0.013 (2)	−0.0006 (18)	0.0011 (19)	0.0013 (19)
C7b	0.009 (2)	0.018 (2)	0.013 (2)	0.0005 (18)	0.0008 (17)	0.0010 (18)
C8b	0.018 (3)	0.015 (3)	0.019 (3)	0.0007 (17)	0.004 (2)	−0.0006 (18)
C9b	0.012 (2)	0.013 (2)	0.014 (2)	0.0019 (17)	0.0001 (18)	0.0003 (18)
C10b	0.011 (2)	0.018 (3)	0.013 (2)	0.0013 (18)	0.0028 (19)	0.0015 (17)
C11b	0.016 (2)	0.014 (2)	0.020 (2)	0.0017 (19)	0.001 (2)	0.0009 (19)

### Geometric parameters (Å, º)

**Table:**

Br1a—C4a	1.906 (5)	C8a—C9a	1.528 (7)
Br1b—C4b	1.904 (5)	C8a—H1c8a	0.96
O1a—C1a	1.352 (6)	C8a—H2c8a	0.96
O1a—H1o1a	0.82 (5)	C9a—H1c9a	0.96
O2a—C11a	1.425 (6)	C9a—H2c9a	0.96
O2a—H1o2a	0.82 (3)	C10a—C11a	1.520 (7)
O1b—C1b	1.345 (6)	C10a—H1c10a	0.96
O1b—H1o1b	0.82 (5)	C10a—H2c10a	0.96
O2b—C11b	1.429 (6)	C11a—H1c11a	0.96
O2b—H1o2b	0.82 (3)	C11a—H2c11a	0.96
N1a—C7a	1.273 (6)	C1b—C2b	1.411 (7)
N1a—C8a	1.460 (6)	C1b—C6b	1.393 (7)
N2a—C9a	1.460 (6)	C2b—C3b	1.411 (7)
N2a—C10a	1.467 (7)	C2b—C7b	1.460 (7)
N2a—H1n2a	0.88 (3)	C3b—C4b	1.387 (7)
N1b—C7b	1.276 (7)	C3b—H1c3b	0.96
N1b—C8b	1.455 (7)	C4b—C5b	1.382 (7)
N2b—C9b	1.461 (6)	C5b—C6b	1.390 (7)
N2b—C10b	1.472 (7)	C5b—H1c5b	0.96
N2b—H1n2b	0.88 (3)	C6b—H1c6b	0.96
C1a—C2a	1.417 (7)	C7b—H1c7b	0.96
C1a—C6a	1.388 (7)	C8b—C9b	1.530 (7)
C2a—C3a	1.400 (7)	C8b—H1c8b	0.96
C2a—C7a	1.465 (7)	C8b—H2c8b	0.96
C3a—C4a	1.384 (7)	C9b—H1c9b	0.96
C3a—H1c3a	0.96	C9b—H2c9b	0.96
C4a—C5a	1.387 (7)	C10b—C11b	1.519 (7)
C5a—C6a	1.387 (7)	C10b—H1c10b	0.96
C5a—H1c5a	0.96	C10b—H2c10b	0.96
C6a—H1c6a	0.96	C11b—H1c11b	0.96
C7a—H1c7a	0.96	C11b—H2c11b	0.96
			
C1a—O1a—H1o1a	112 (4)	O2a—C11a—C10a	111.3 (4)
C11a—O2a—H1o2a	101 (5)	O2a—C11a—H1c11a	109.47
C1b—O1b—H1o1b	115 (4)	O2a—C11a—H2c11a	109.47
C11b—O2b—H1o2b	105 (5)	C10a—C11a—H1c11a	109.47
C7a—N1a—C8a	118.0 (4)	C10a—C11a—H2c11a	109.47
C9a—N2a—C10a	111.6 (4)	H1c11a—C11a—H2c11a	107.54
C9a—N2a—H1n2a	113 (4)	O1b—C1b—C2b	121.2 (4)
C10a—N2a—H1n2a	109 (4)	O1b—C1b—C6b	119.1 (4)
C7b—N1b—C8b	117.9 (4)	C2b—C1b—C6b	119.7 (5)
C9b—N2b—C10b	112.2 (4)	C1b—C2b—C3b	119.1 (4)
C9b—N2b—H1n2b	108 (4)	C1b—C2b—C7b	120.6 (4)
C10b—N2b—H1n2b	105 (4)	C3b—C2b—C7b	119.6 (4)
O1a—C1a—C2a	121.3 (4)	C2b—C3b—C4b	119.5 (4)
O1a—C1a—C6a	119.5 (4)	C2b—C3b—H1c3b	120.24
C2a—C1a—C6a	119.2 (4)	C4b—C3b—H1c3b	120.24
C1a—C2a—C3a	119.4 (4)	Br1b—C4b—C3b	118.6 (4)
C1a—C2a—C7a	120.9 (4)	Br1b—C4b—C5b	119.7 (4)
C3a—C2a—C7a	119.4 (4)	C3b—C4b—C5b	121.5 (4)
C2a—C3a—C4a	119.5 (4)	C4b—C5b—C6b	119.4 (4)
C2a—C3a—H1c3a	120.23	C4b—C5b—H1c5b	120.31
C4a—C3a—H1c3a	120.23	C6b—C5b—H1c5b	120.31
Br1a—C4a—C3a	118.9 (4)	C1b—C6b—C5b	120.8 (4)
Br1a—C4a—C5a	119.3 (4)	C1b—C6b—H1c6b	119.6
C3a—C4a—C5a	121.7 (4)	C5b—C6b—H1c6b	119.6
C4a—C5a—C6a	118.8 (4)	N1b—C7b—C2b	121.7 (4)
C4a—C5a—H1c5a	120.6	N1b—C7b—H1c7b	119.13
C6a—C5a—H1c5a	120.6	C2b—C7b—H1c7b	119.13
C1a—C6a—C5a	121.4 (5)	N1b—C8b—C9b	112.0 (4)
C1a—C6a—H1c6a	119.31	N1b—C8b—H1c8b	109.47
C5a—C6a—H1c6a	119.31	N1b—C8b—H2c8b	109.47
N1a—C7a—C2a	121.5 (4)	C9b—C8b—H1c8b	109.47
N1a—C7a—H1c7a	119.24	C9b—C8b—H2c8b	109.47
C2a—C7a—H1c7a	119.24	H1c8b—C8b—H2c8b	106.79
N1a—C8a—C9a	109.3 (4)	N2b—C9b—C8b	109.5 (4)
N1a—C8a—H1c8a	109.47	N2b—C9b—H1c9b	109.47
N1a—C8a—H2c8a	109.47	N2b—C9b—H2c9b	109.47
C9a—C8a—H1c8a	109.47	C8b—C9b—H1c9b	109.47
C9a—C8a—H2c8a	109.47	C8b—C9b—H2c9b	109.47
H1c8a—C8a—H2c8a	109.64	H1c9b—C9b—H2c9b	109.45
N2a—C9a—C8a	111.0 (4)	N2b—C10b—C11b	109.7 (4)
N2a—C9a—H1c9a	109.47	N2b—C10b—H1c10b	109.47
N2a—C9a—H2c9a	109.47	N2b—C10b—H2c10b	109.47
C8a—C9a—H1c9a	109.47	C11b—C10b—H1c10b	109.47
C8a—C9a—H2c9a	109.47	C11b—C10b—H2c10b	109.47
H1c9a—C9a—H2c9a	107.95	H1c10b—C10b—H2c10b	109.22
N2a—C10a—C11a	110.8 (4)	O2b—C11b—C10b	111.6 (4)
N2a—C10a—H1c10a	109.47	O2b—C11b—H1c11b	109.47
N2a—C10a—H2c10a	109.47	O2b—C11b—H2c11b	109.47
C11a—C10a—H1c10a	109.47	C10b—C11b—H1c11b	109.47
C11a—C10a—H2c10a	109.47	C10b—C11b—H2c11b	109.47
H1c10a—C10a—H2c10a	108.1	H1c11b—C11b—H2c11b	107.25

### Hydrogen-bond geometry (Å, º)

**Table:**

*D*—H···*A*	*D*—H	H···*A*	*D*···*A*	*D*—H···*A*
O1*a*—H1*o*1*a*···N1*a*	0.82 (5)	1.89 (6)	2.597 (5)	144 (5)
O1*a*—H1*o*1*a*···C7*a*	0.82 (5)	2.45 (6)	2.876 (6)	113 (4)
O2*a*—H1*o*2*a*···N2*b*^i^	0.82 (3)	2.02 (4)	2.826 (6)	168 (6)
O1*b*—H1*o*1*b*···N1*b*	0.82 (5)	1.91 (6)	2.587 (6)	139 (5)
O2*b*—H1*o*2*b*···N2*a*^ii^	0.82 (3)	2.06 (4)	2.859 (6)	165 (6)
N2*a*—H1*n*2*a*···O2*a*	0.88 (3)	2.45 (5)	2.871 (6)	110 (5)
N2*b*—H1*n*2*b*···O2*b*	0.88 (3)	2.44 (5)	2.884 (5)	112 (5)

Symmetry codes: (i) −*x*+2, *y*−1/2, −*z*+2; (ii) −*x*+1, *y*+1/2, −*z*+1.
